# Supplementary figures and images for: Examining the overlap in lymphatic filariasis prevalence and malaria insecticide-treated net access-use in endemic Africa
Source: PLoS Negl Trop Dis. 2025 Jun 26;19(6):e0013165. doi: 10.1371/journal.pntd.0013165 (PMC12201664; doi:10.1371/journal.pntd.0013165)

LF prevalence 2018 (%) and  
IRS use 2020 (%)

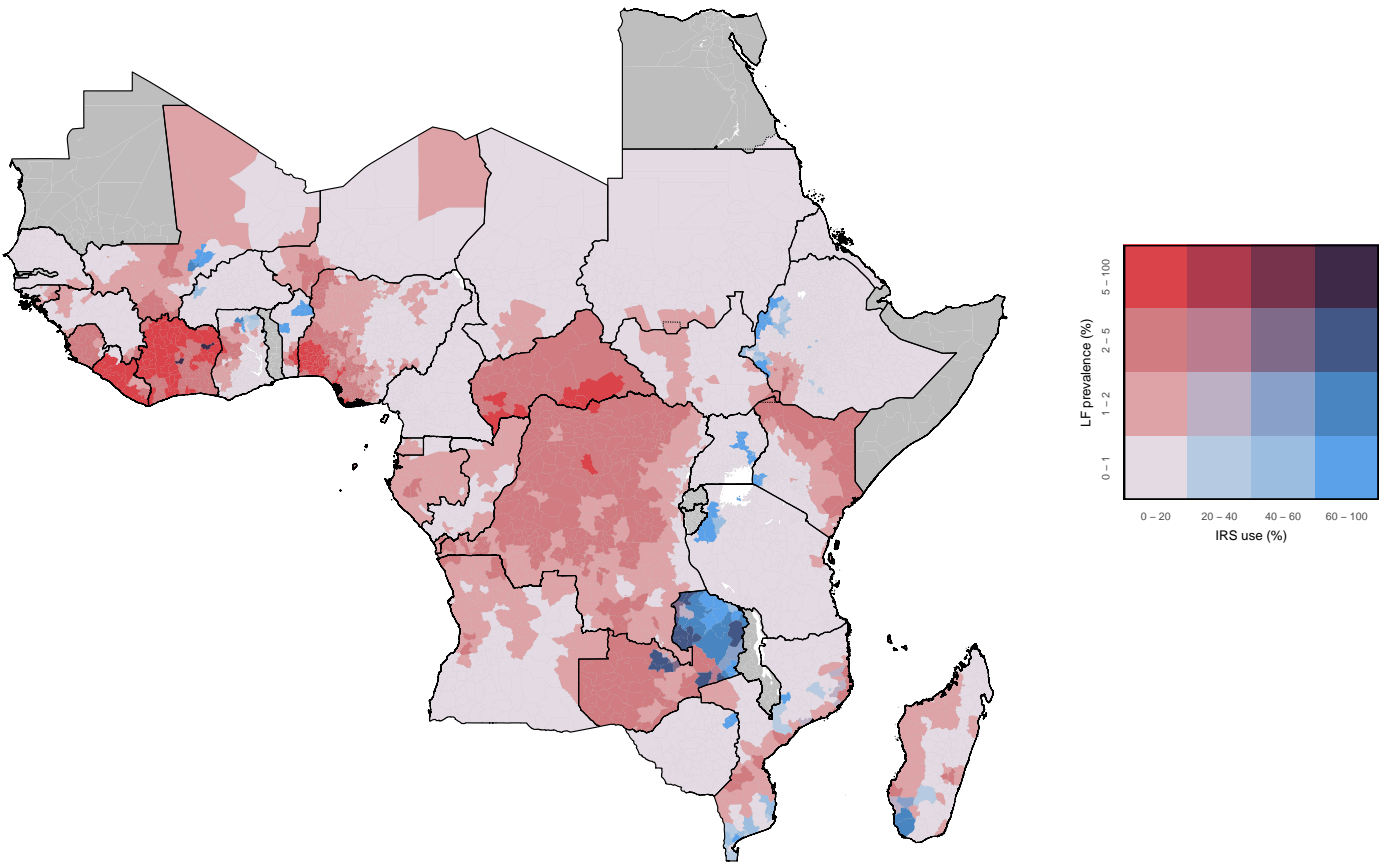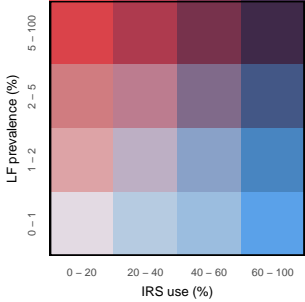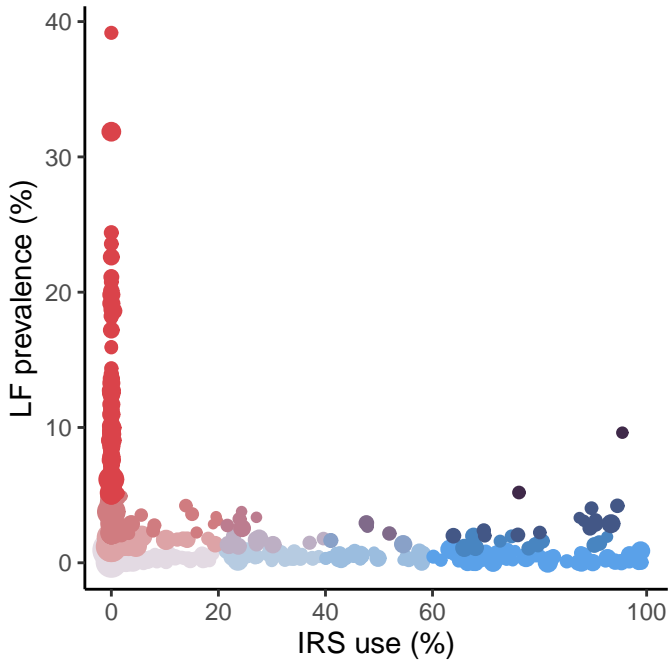

Supplement: S1 Fig — The bivariate choropleth map and scatter plot color key in the center indicate the degree to which LF prevalence (vertical axis, white to red) and IRS use (horizontal axis, white to blue) overlap. Grey indicates areas considered to be non-endemic. LF: lymphatic filariasis; IRS: indoor residual spraying. Map base layer shapefile is from ESPEN, available from: https://espen.afro.who.int/tools-resources/data-query-tools/cartography-database [18]. (PDF) [file pntd.0013165.s001.pdf]

LF prevalence 2018 (%) and  
IRS use 2020 (%)

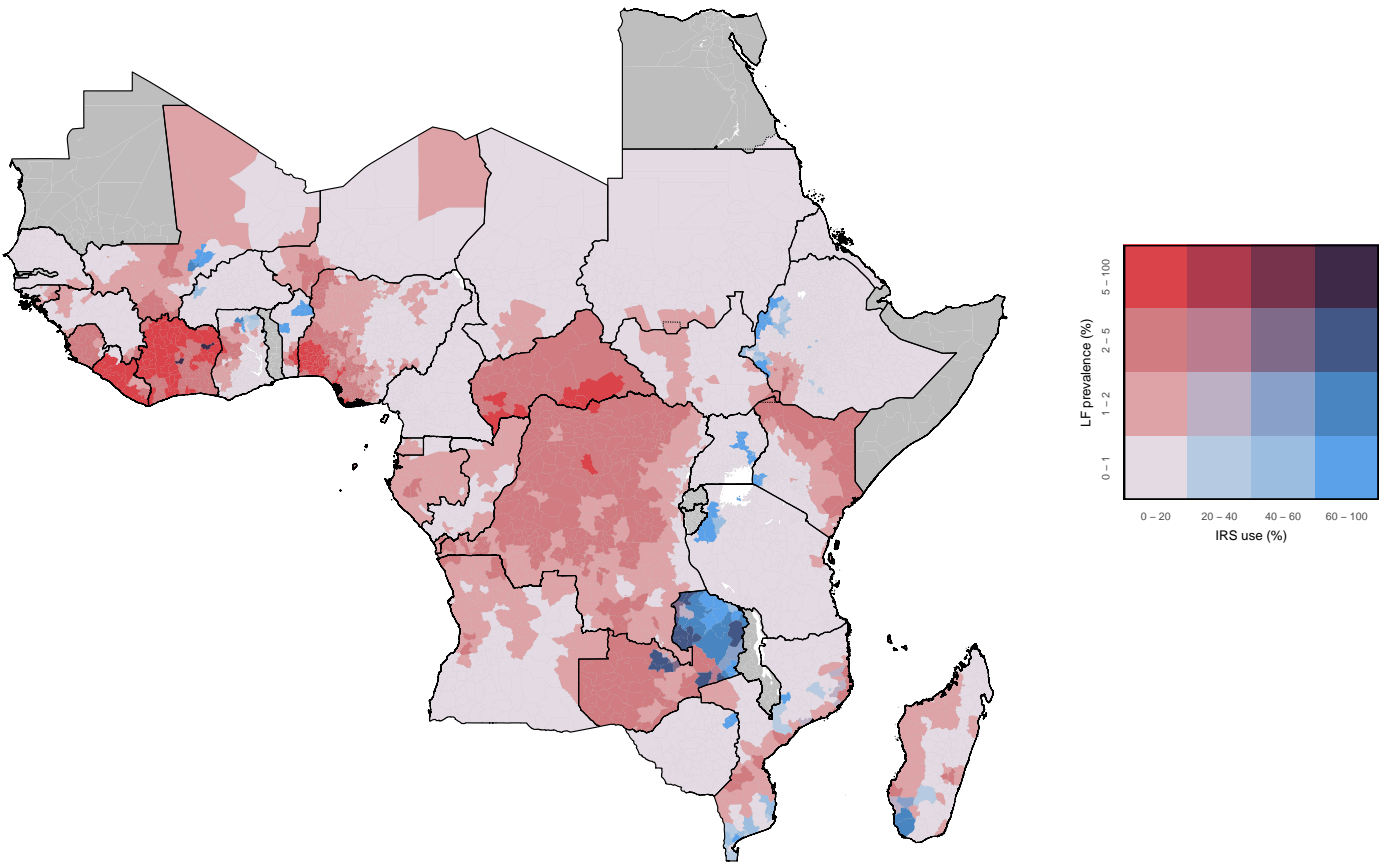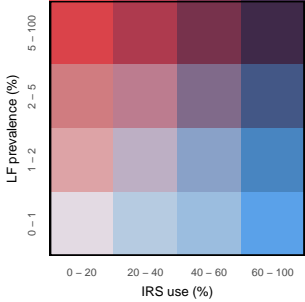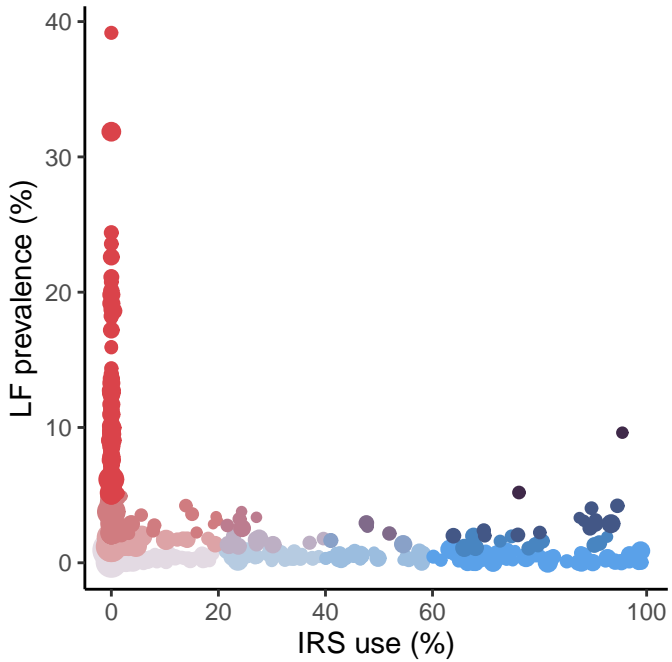

Supplement: S2 Fig — The bivariate choropleth map and scatter plot color key in the center indicate the degree to which LF prevalence (vertical axis, white to red) and IRS use (horizontal axis, white to blue) overlap. Grey indicates areas considered to be non-endemic. LF: lymphatic filariasis; IRS: indoor residual spraying. Map base layer shapefile is from ESPEN, available from: https://espen.afro.who.int/tools-resources/data-query-tools/cartography-database [18]. (PDF) [file pntd.0013165.s002.pdf]

LF prevalence 2018 (%) and  
Malaria prevalence 2019 (%)

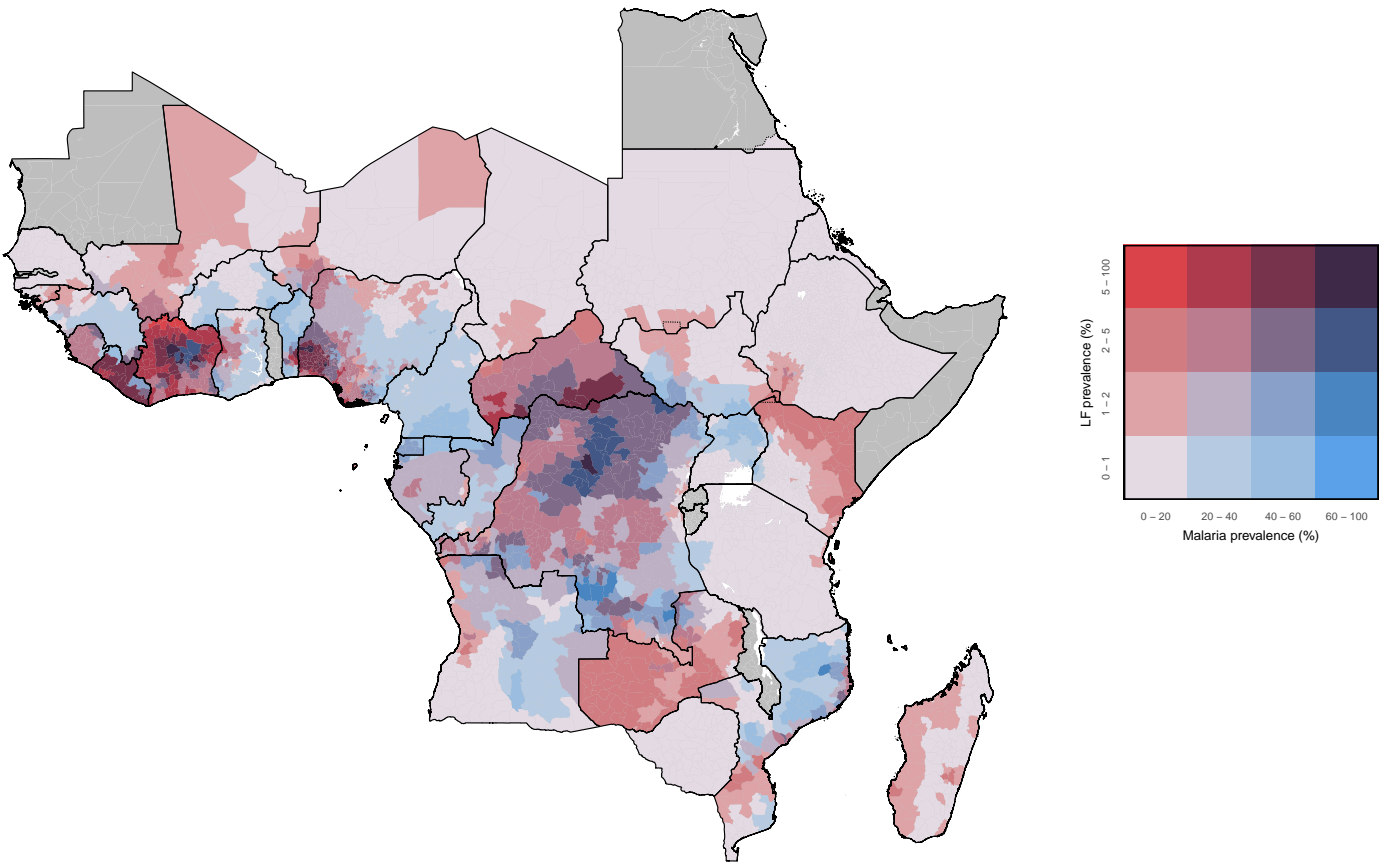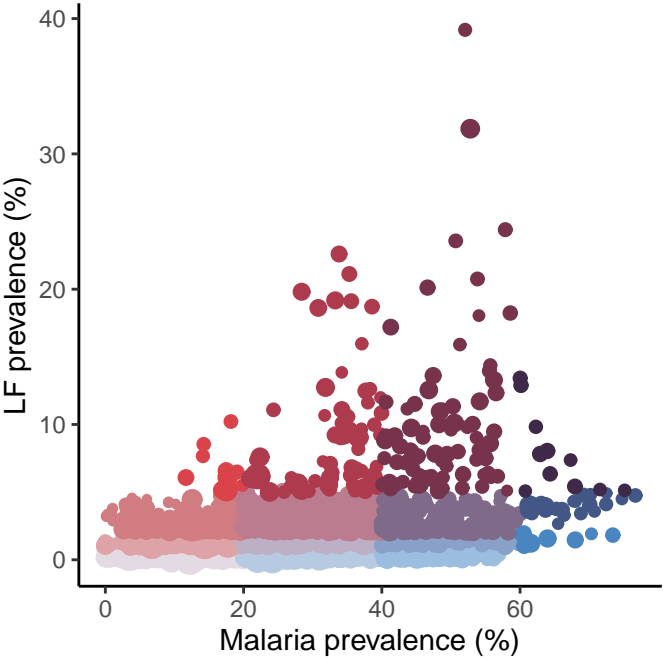

Supplement: S3 Fig — The bivariate choropleth map and scatter plot color key in the center indicate the degree to which LF prevalence (vertical axis, white to red) and malaria Pf prevalence (horizontal axis, white to blue) overlap. Grey indicates areas considered to be non-endemic. LF: lymphatic filariasis; Pf: Plasmodium falciparum. Map base layer shapefile is from ESPEN, available from: https://espen.afro.who.int/tools-resources/data-query-tools/cartography-database [18]. (PDF) [file pntd.0013165.s003.pdf]

LF prevalence 2018 (%) and  
ITN access 2018 (%)

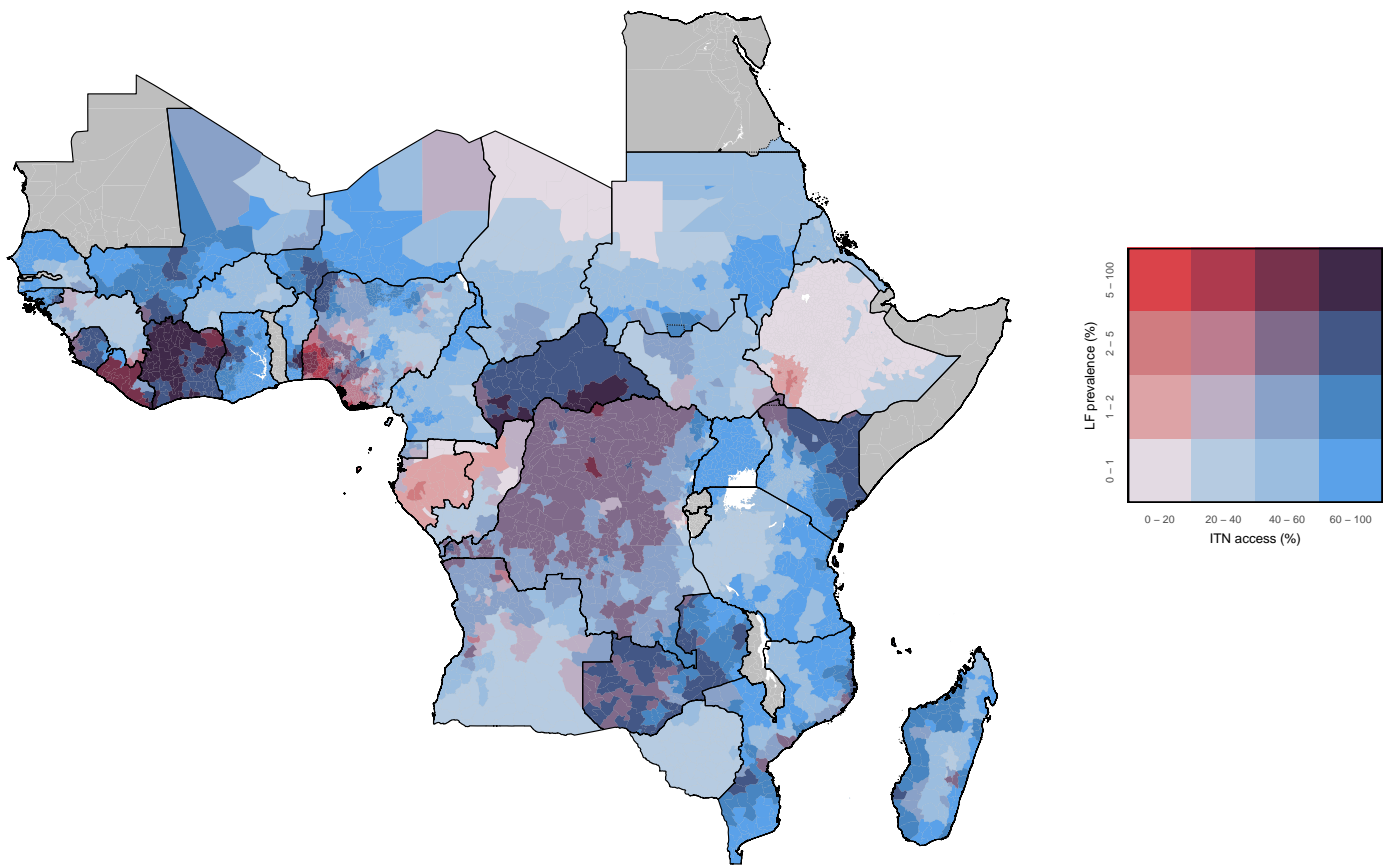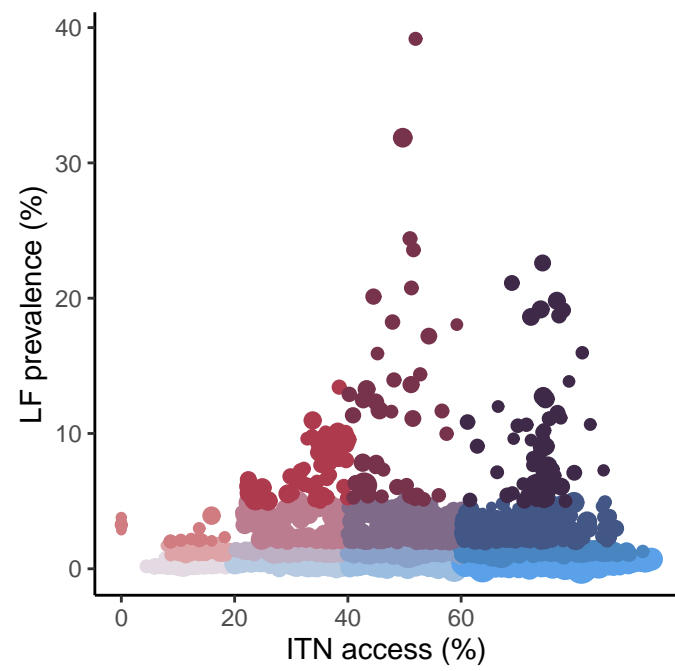

Supplement: S4 Fig — The bivariate choropleth map and scatter plot color key in the center indicate the degree to which LF prevalence (vertical axis, white to red) and ITN access (horizontal axis, white to blue) overlap. Grey indicates areas considered to be non-endemic. LF: lymphatic filariasis; ITN: insecticide-treated nets. Map base layer shapefile is from ESPEN, available from: https://espen.afro.who.int/tools-resources/data-query-tools/cartography-database [18]. (PDF) [file pntd.0013165.s004.pdf]

LF prevalence 2018 (counts) and ITN access 2018 (%)

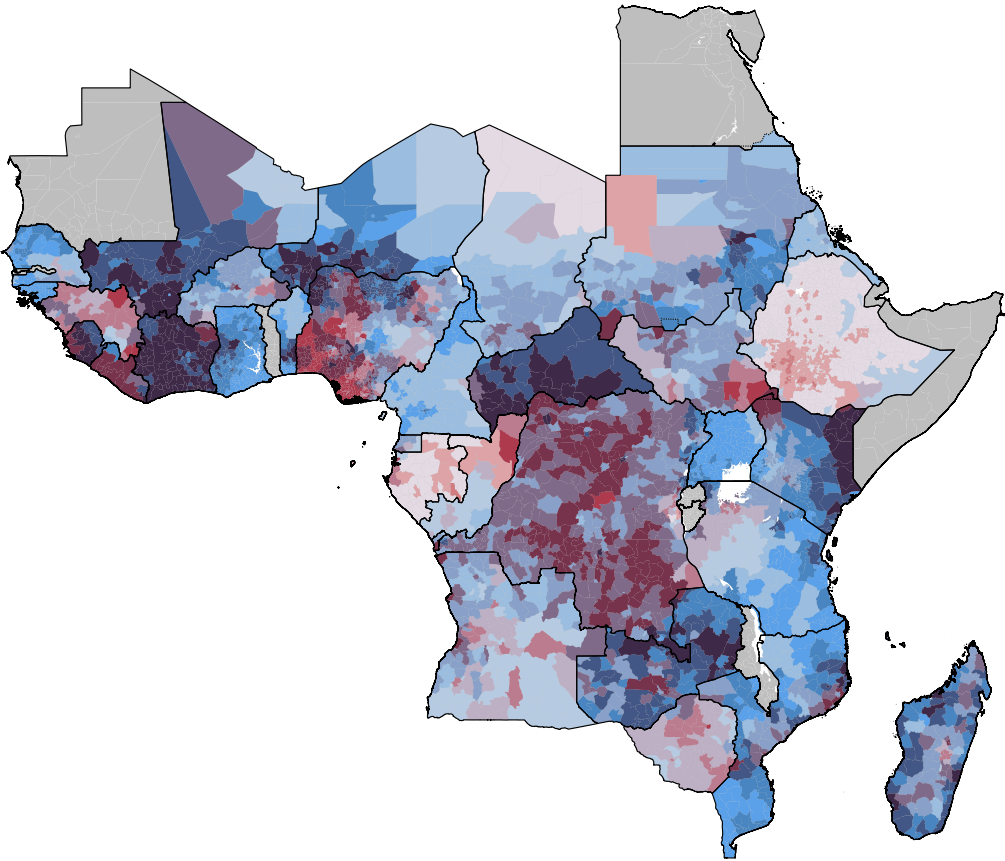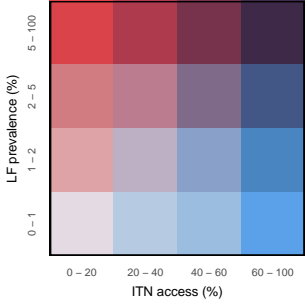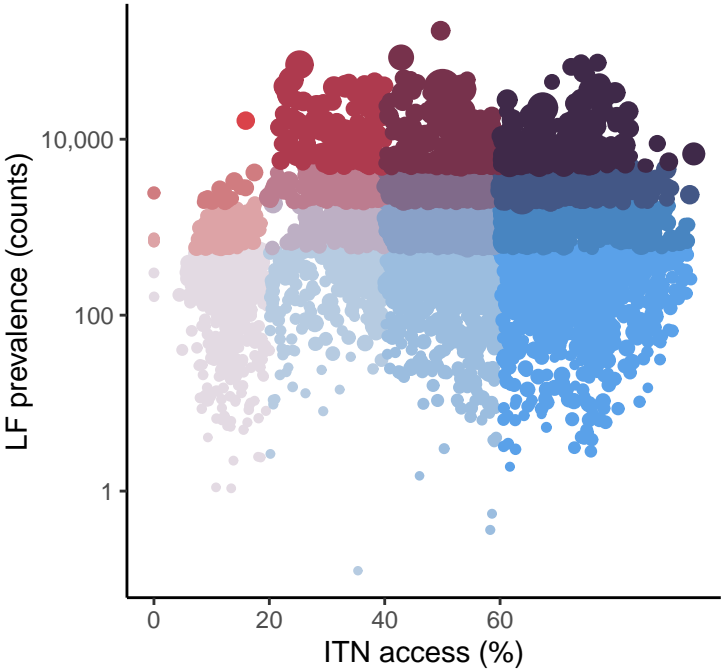

Supplement: S5 Fig — The bivariate choropleth map and scatter plot color key in the center indicate the degree to which LF prevalence (vertical axis, white to red) and ITN access (horizontal axis, white to blue) overlap. Grey indicates areas considered to be non-endemic. LF: lymphatic filariasis; ITN: insecticide-treated nets. Map base layer shapefile is from ESPEN, available from: https://espen.afro.who.int/tools-resources/data-query-tools/cartography-database [18]. (PDF) [file pntd.0013165.s005.pdf]

LF prevalence 2018 (%) and  
ITN use 2018 (%)

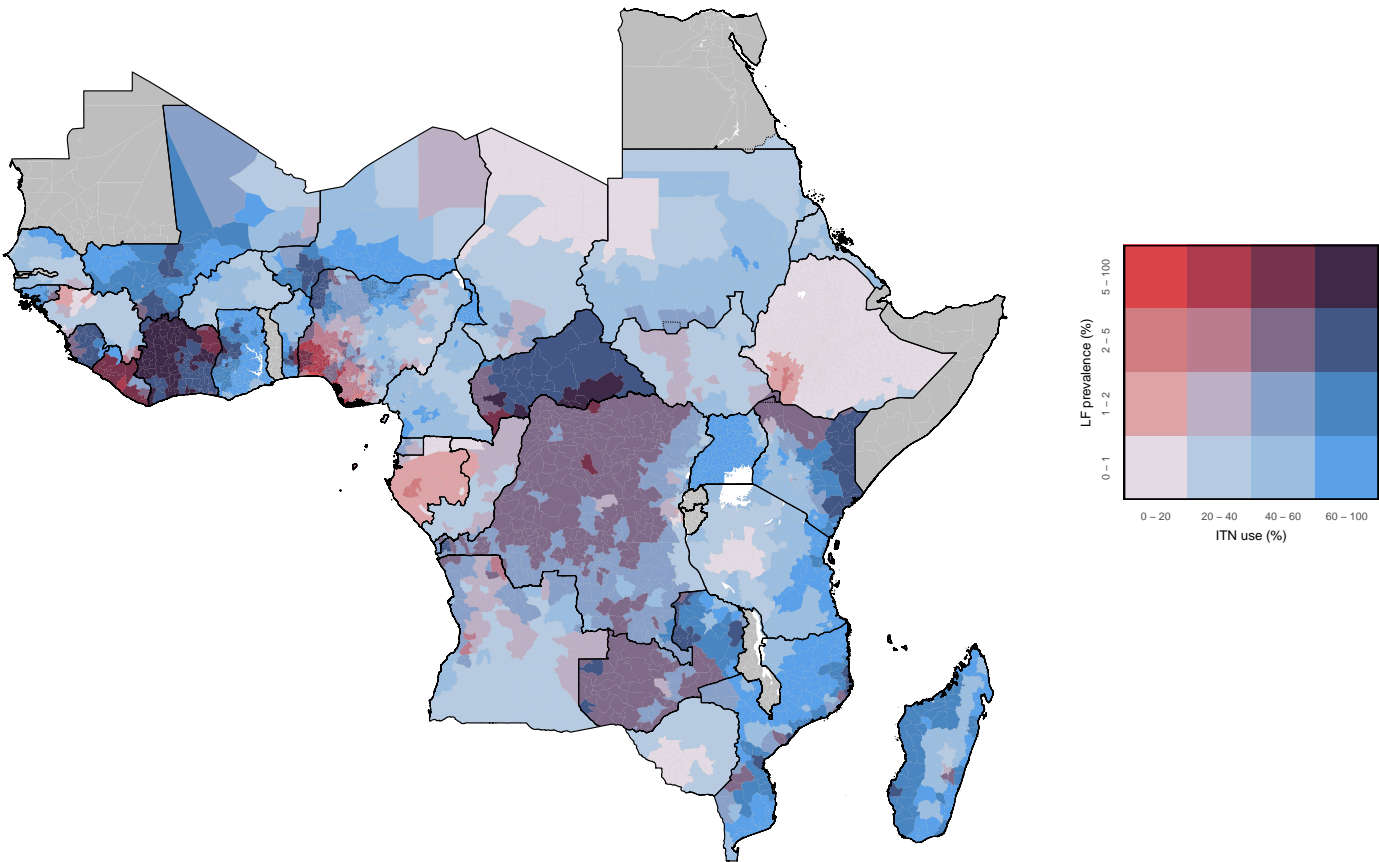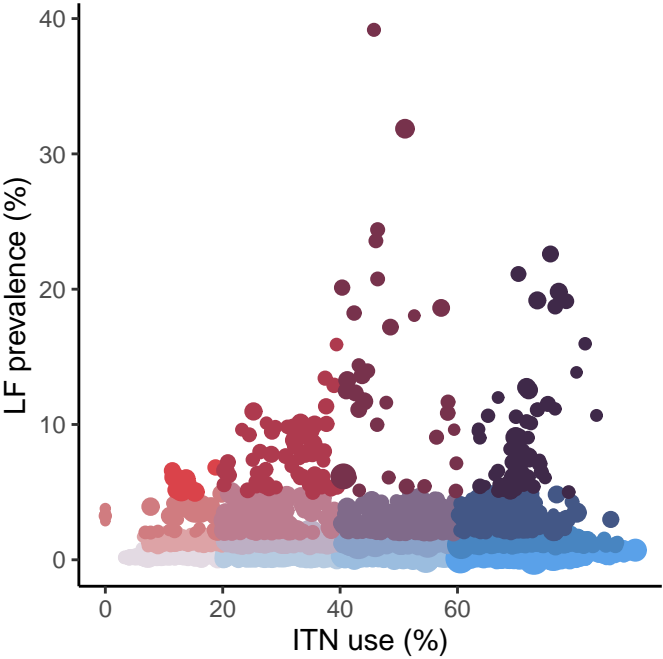

Supplement: S6 Fig — The bivariate choropleth map and scatter plot color key in the center indicate the degree to which LF prevalence (vertical axis, white to red) and ITN use (horizontal axis, white to blue) overlap. Grey indicates areas considered to be non-endemic. LF: lymphatic filariasis; ITN: insecticide-treated nets. Map base layer shapefile is from ESPEN, available from: https://espen.afro.who.int/tools-resources/data-query-tools/cartography-database [18]. (PDF) [file pntd.0013165.s006.pdf]

LF prevalence 2018 (counts) and  
ITN use 2018 (%)

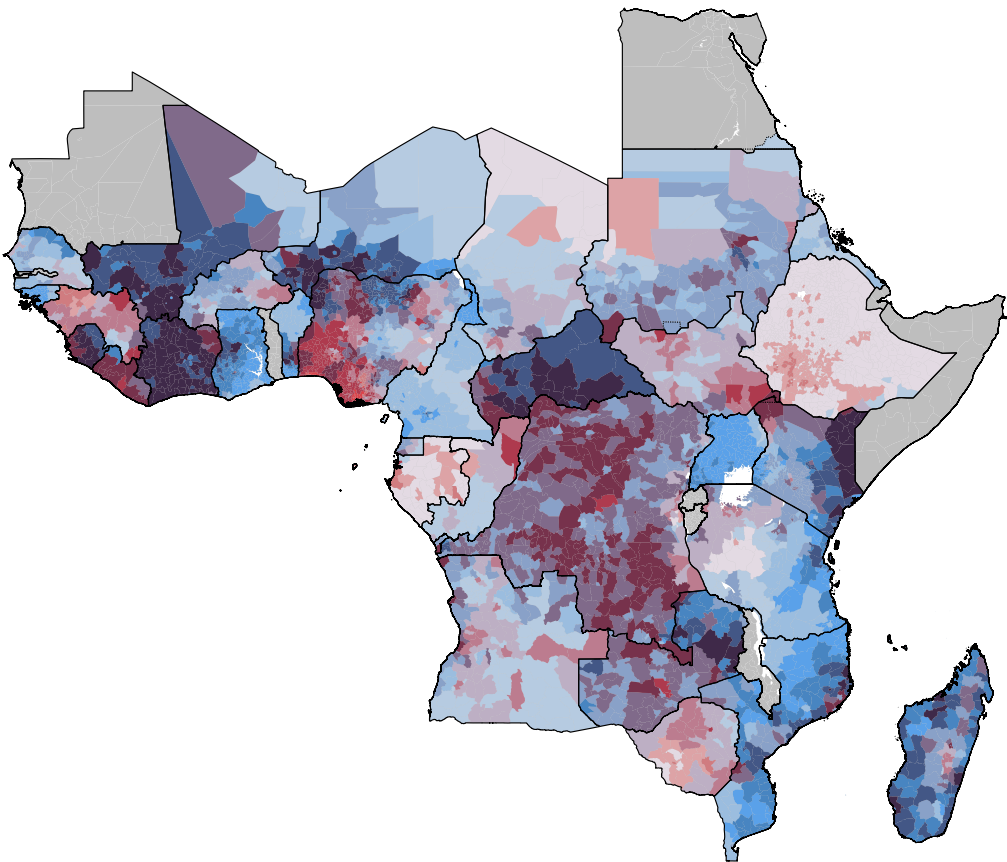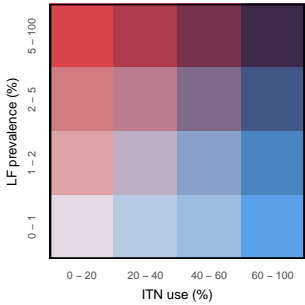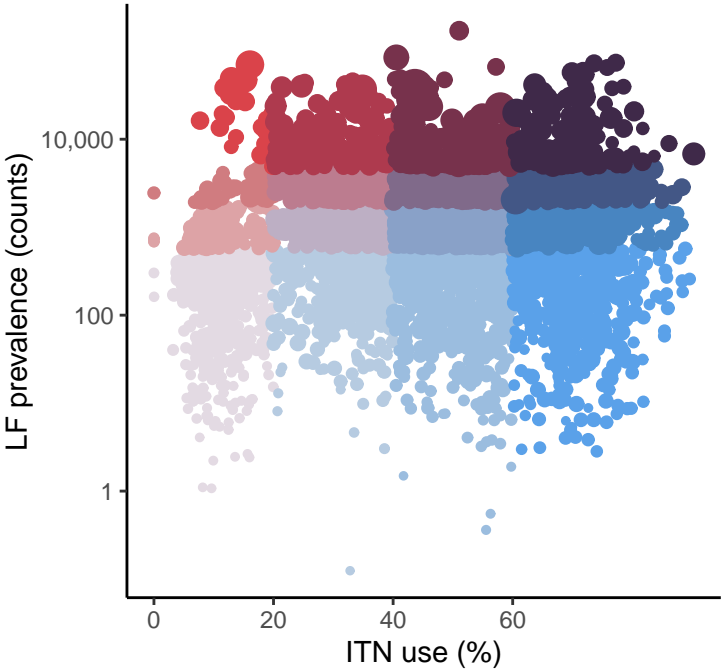

Supplement: S7 Fig — The bivariate choropleth map and scatter plot color key in the center indicate the degree to which LF prevalence (vertical axis, white to red) and ITN use (horizontal axis, white to blue) overlap. Grey indicates areas considered to be non-endemic. LF: lymphatic filariasis; ITN: insecticide-treated nets. Map base layer shapefile is from ESPEN, available from: https://espen.afro.who.int/tools-resources/data-query-tools/cartography-database [18]. (PDF) [file pntd.0013165.s007.pdf]

LF prevalence 2018 (%) and  
IRS use 2018 (%)

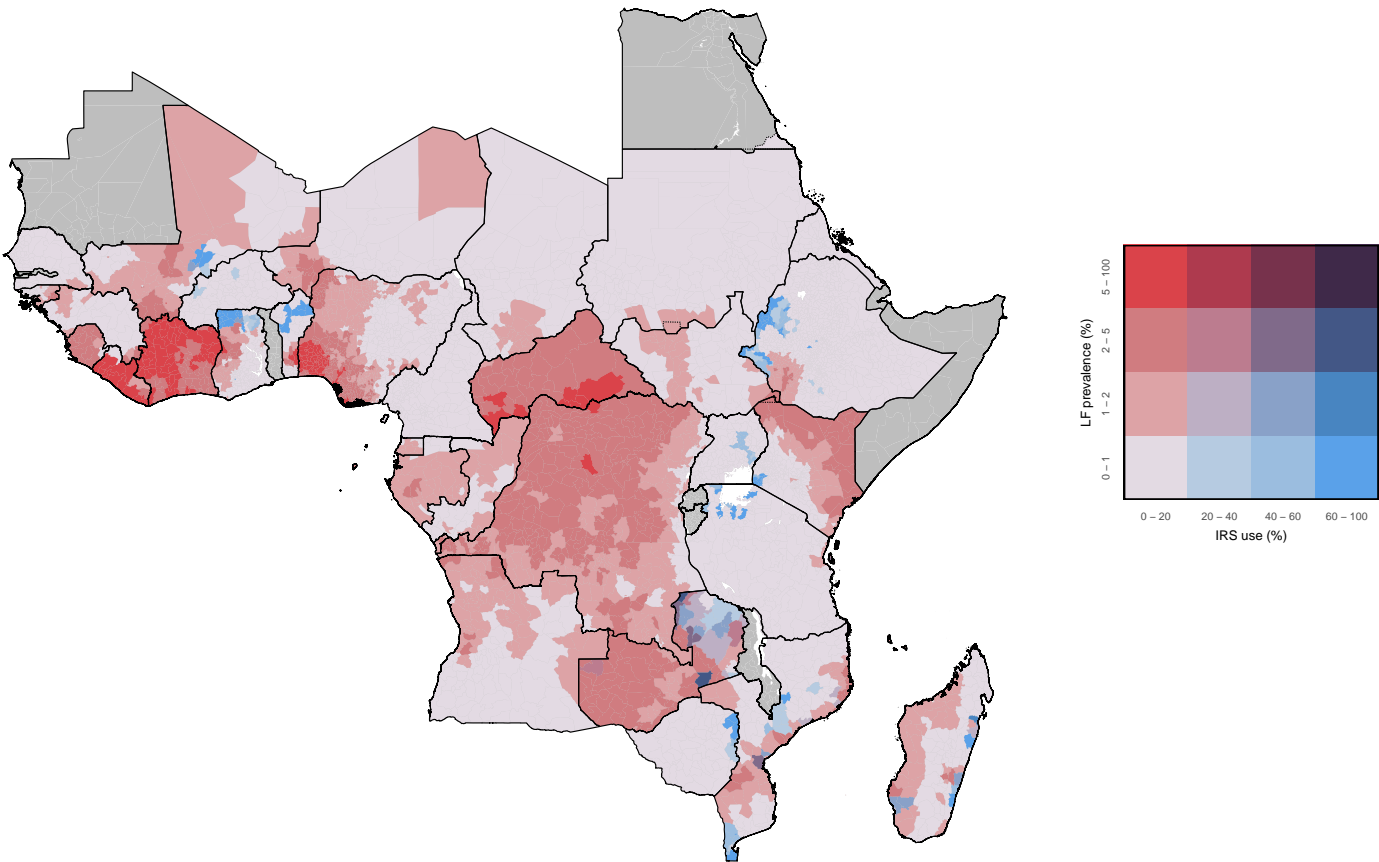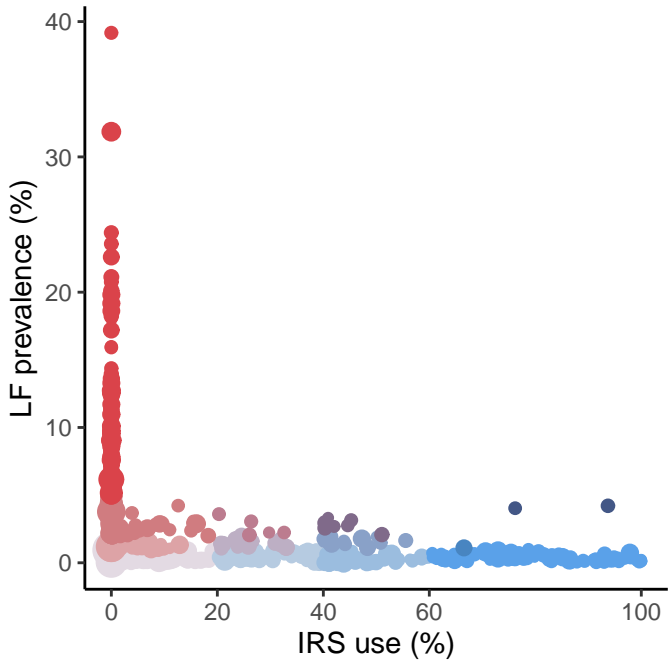

Supplement: S8 Fig — The bivariate choropleth map and scatter plot color key in the center indicate the degree to which LF prevalence (vertical axis, white to red) and IRS use (horizontal axis, white to blue) overlap. Grey indicates areas considered to be non-endemic. LF: lymphatic filariasis; IRS: indoor residual spraying. Map base layer shapefile is from ESPEN, available from: https://espen.afro.who.int/tools-resources/data-query-tools/cartography-database [18]. (PDF) [file pntd.0013165.s008.pdf]

LF prevalence 2018 (counts) and  
IRS use 2018 (%)

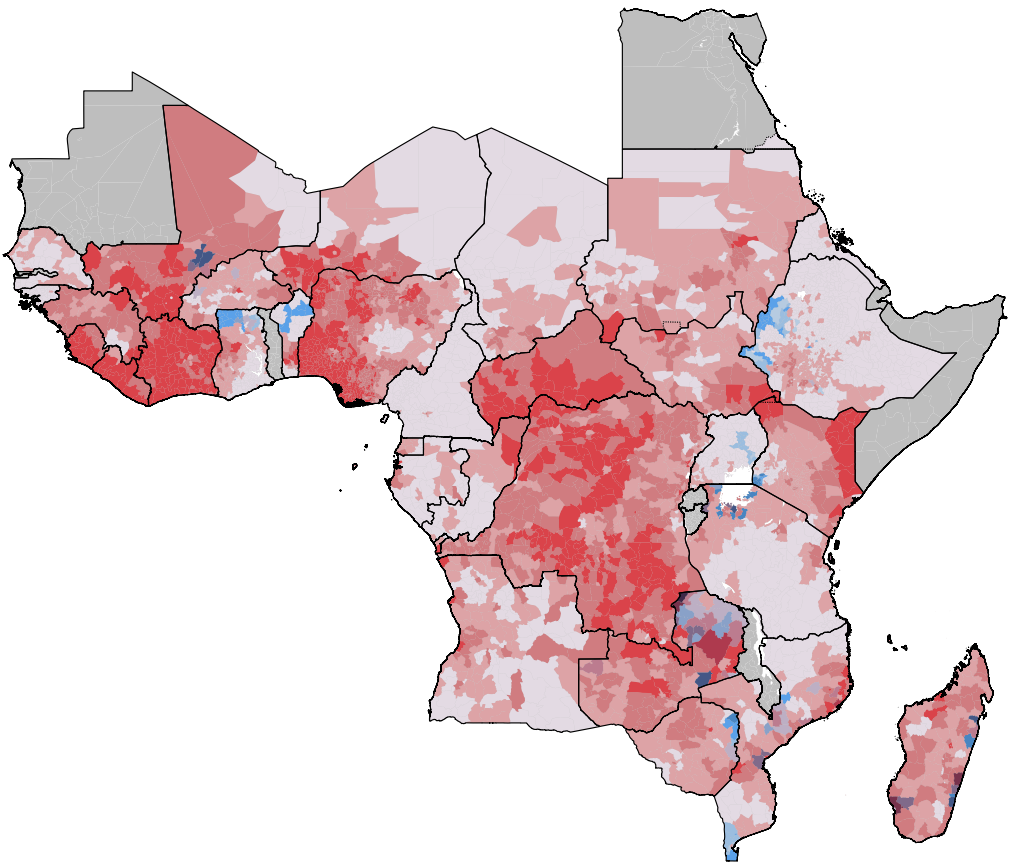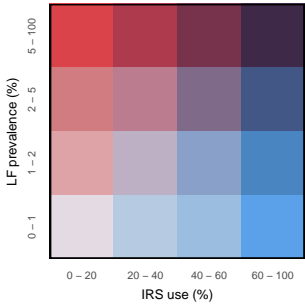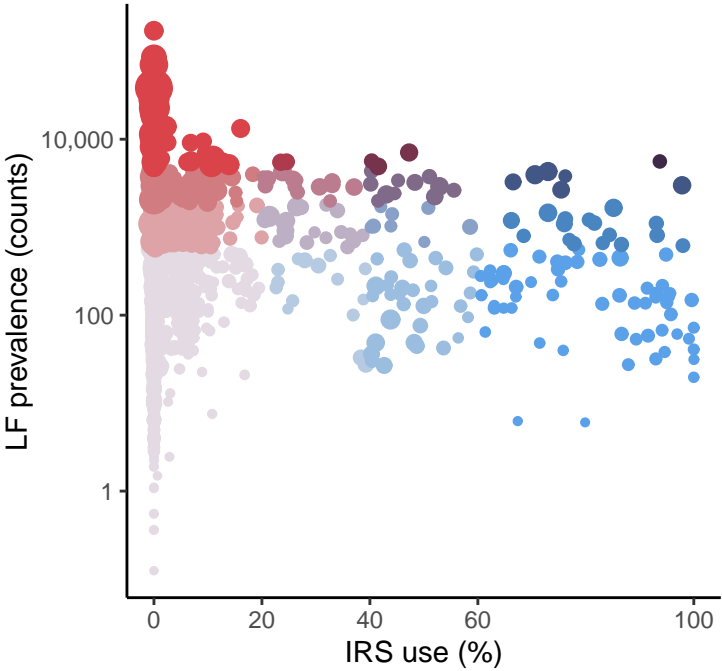

Supplement: S9 Fig — The bivariate choropleth map and scatter plot color key in the center indicate the degree to which LF prevalence (vertical axis, white to red) and IRS use (horizontal axis, white to blue) overlap. Grey indicates areas considered to be non-endemic. LF: lymphatic filariasis; IRS: indoor residual spraying. Map base layer shapefile is from ESPEN, available from: https://espen.afro.who.int/tools-resources/data-query-tools/cartography-database [18]. (PDF) [file pntd.0013165.s009.pdf]

LF prevalence 2018 (%) and  
Malaria prevalence 2018 (%)

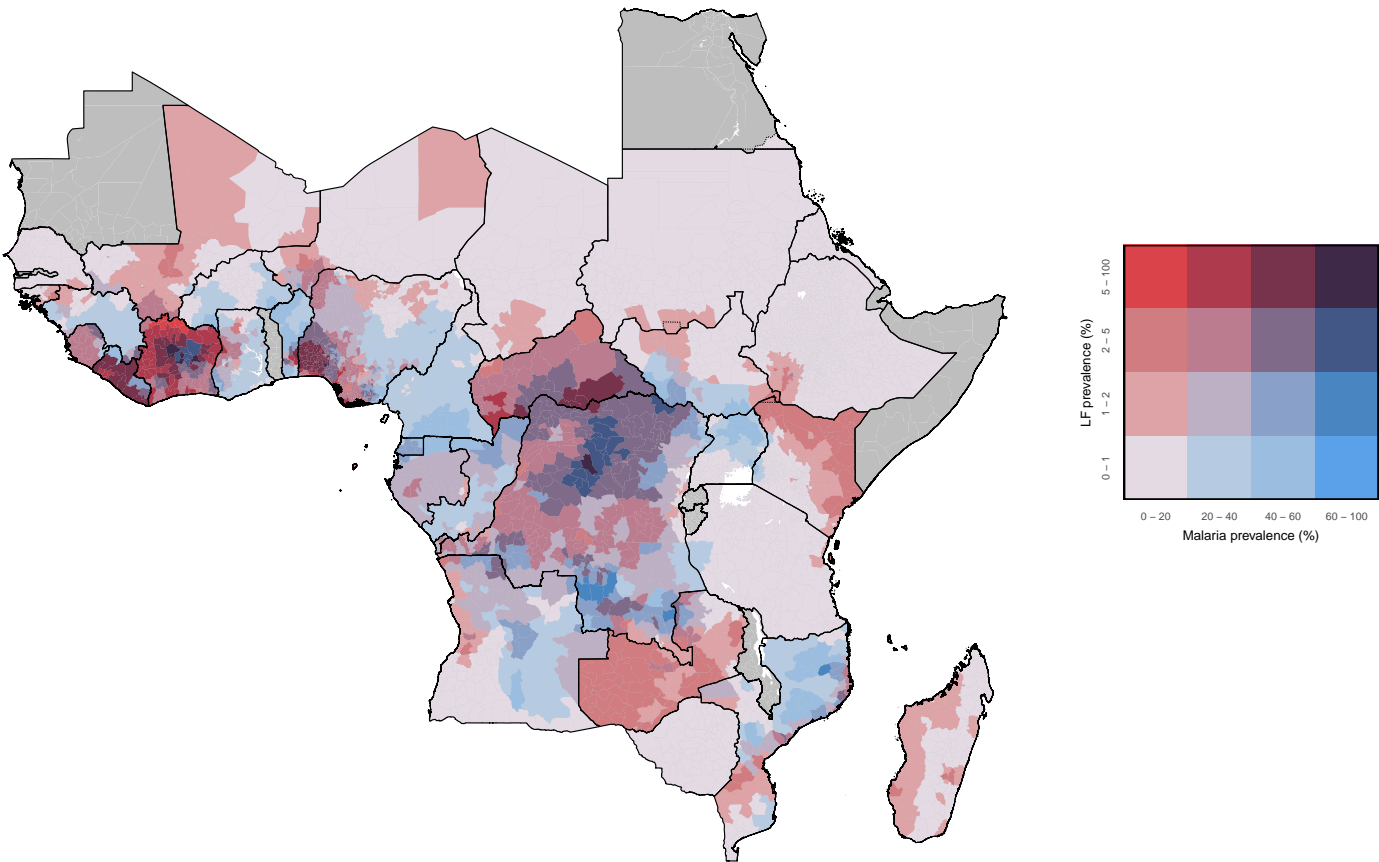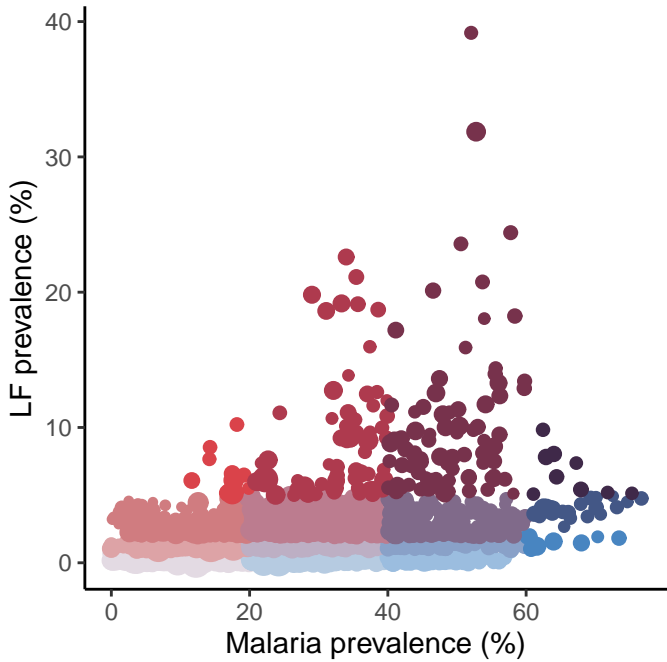

Supplement: S10 Fig — The bivariate choropleth map and scatter plot color key in the center indicate the degree to which LF prevalence (vertical axis, white to red) and malaria Pf prevalence (horizontal axis, white to blue) overlap. Grey indicates areas considered to be non-endemic. LF: lymphatic filariasis; Pf: Plasmodium falciparum. Map base layer shapefile is from ESPEN, available from: https://espen.afro.who.int/tools-resources/data-query-tools/cartography-database [18]. (PDF) [file pntd.0013165.s010.pdf]
