# Supplementary material for: Examining the overlap in lymphatic filariasis prevalence and malaria insecticide-treated net access-use in endemic Africa
Source: PLoS Negl Trop Dis. 2025 Jun 26;19(6):e0013165. doi: 10.1371/journal.pntd.0013165 (PMC12201664; doi:10.1371/journal.pntd.0013165)
Supplement: Attachment — Submitted filename: Response to Reviewers_final.docx [file pntd.0013165.s014.docx]

**PLOS NTDs paper revisions 2025-01-13**

Author comments are in blue.

Line numbers referenced refer to the lines in the tracked changes version of the manuscript once all revisions have been accepted.

**Methods**
 -Are the objectives of the study clearly articulated with a clear testable hypothesis stated?

-Is the study design appropriate to address the stated objectives?

-Is the population clearly described and appropriate for the hypothesis being tested?

-Is the sample size sufficient to ensure adequate power to address the hypothesis being tested?

-Were correct statistical analysis used to support conclusions?

-Are there concerns about ethical or regulatory requirements being met?

Reviewer #1: I am unable to assess the statistical and geospatial analysis in the paper. Other aspects of the methodology are appropriately described.

We thank the reviewer for their kind comments.

Reviewer #2: The objectives are clearly stated. The paper aims to overlay published geospatial estimates of ITN access and use, and LF prevalence estimates at 33 LF and malaria endemic locations in Africa. The paper then looks at the ITN coverage in high and low LF prevalence areas to assess how effective malaria control programs have been in providing access and use to ITNs that would potentially contribute to interruption of LF. The study design is appropriate to address this since these spatial datasets are available through the extensive prior published work on the three aspects studied (ITNs access and use, LF prevalence spatial prediction, Malaria prevalence spatial prediction PfPR) that has been done by this group and many others, together with the decades of work of surveyors and national programmes who provided the raw data to the spatial modelers of the three aspects. I appreciated being provided with the prior publications of these parameters as well as IRS coverage. Putting these three predictions together is a valid and useful approach to visualizing and assessing the overlap in ITN access-use and LF/malaria prevalence. The explanation for which year has been chosen for the overlap is clear and reasonable (lines 136 -137). However, there are some gaps in 1) how the results are explained 2) evaluating the implications of the findings for future control. There is no specific hypothesis about what would have been expected as far as I could tell.

We thank the reviewer for their complimentary comments. Please see responses regarding the gaps mentioned in relevant sections below.

Given that the LF prevalence estimates are produced by 5 km2 grid (Local Burden of Disease collaborators, 2019) like the ITN and PfPR, why did you choose to aggregate the estimates into Implementation units (line 147)? What is the usual size of an IU (district or other unit?) and how much does it vary by country?

We thank the reviewer for the opportunity to clarify and add additional text defining IUs and our rationale for using them (lines 145-151). We chose to present our results at the implementation unit level as it is the level that many programmatic decisions are made regarding lymphatic filariasis control. We expect that this unit of aggregation would therefore be the most useful for policy makers who may be interested in the relationship between a programmatic intervention (ITN use) and LF prevalence. IUs represent the administrative units used for implementing LF interventions within a country; while this is typically the second administrative level (ie, district), there can be variation between countries depending on the structure and objectives of each country’s LF control program. (Please see page 3 of: <https://iris.who.int/handle/10665/69172>)

Additional text:

Using population estimates from WorldPop (17), we aggregated the 5x5 km^2^ estimates of LF prevalence, ITN use, ITN access, malaria prevalence, IRS use, and population to the Expanded Special Project for Elimination of Neglected Tropical Diseases (ESPEN) administrative implementing units (IU) level using fractional aggregation, for 33 LF and malaria-endemic locations in Africa, and then overlaid the IU-level aggregates (18). IUs represent the administrative units designated by a country to be used for intervention implementation (19). While these are typically at the district level, there may be variation between countries depending on the structure and objectives of each country’s control program (19).

**Results**

-Does the analysis presented match the analysis plan?

-Are the results clearly and completely presented?

-Are the figures (Tables, Images) of sufficient quality for clarity?

Reviewer #1: Yes.

We thank the reviewer for their comment.

Reviewer #2: Presentation of results is sometimes very confusing. Definitions need to be very clear and cutoffs (e.g. low' or high' ITN "coverage") justified.

We thank the reviewer for their comment. Edits clarifying the cutoffs used and the rationale for their selection have been made on lines 166-168:

Previous text:

For ITN coverage (access and use) and malaria prevalence we used the following definitions: lowest <20%; low 20-39.9%; high 40%-59.9%; highest ≥60%.

Revised text:

In the absence of well-established standard classification thresholds, we categorized ITN access, use, and malaria prevalence using the following definitions based on the IU-level distributions of these metrics: lowest (<20%); low (20-<40%); high (40%-<60%); highest (≥60%).

The first issue I had is with the definition of ITN 'coverage'. It is unfortunate that this word has been used to refer to a composite of access and use "where use among the total population and access among the total population were both separately above or below a specified threshold" line 160. ITN coverage was traditionally defined at the household level by WHO and in DHS surveys ("Proportion of HH with at least 1 net"). I understand that this is likely now out of date, as is the household rather than individual definition of access, and better measures such as nets per capita are becoming more common (as defined clearly in the Bertozzi-Villa 2021 paper). Nevertheless it might have been better to coin another term rather than 'coverage' to avoid confusion. Also, a composite measure of two parameters seems less than optimal when one (use) depends on the other (access). I understand the desire for one measure, but this one seems overcomplicated to me.

We thank the reviewer for this comment and opportunity to clarify in the text. We appreciate the potential for confusion and have more clearly defined the terms (lines 156-159) to better align with the Bertozzi-Villa 2021 paper. Following this, we no longer use “coverage” to refer to any quantitative metrics but instead refer only to access and use (lines 215-221, and 237-239), and use “coverage” only to refer to the conceptual combination of both.

Previous text (lines 156-159):

We defined ITN coverage as IUs where use among the total population, and access among the total population were both separately above or below a specified threshold.

Revised text (lines 156-159):

We use two ITN metrics in this analysis: access, or the proportion of people among the total population who have access to an ITN; and use, or the proportion of the total population that use an ITN. Following Bertozzi-Villa et al., 2021 (14) we refer to specific metrics, like access and use, by name, and use coverage to more generally refer to combinations of metrics.

Previous text (lines 215-221):

Approximately 53.5% (93/174) of high LF prevalence areas were also high prevalence for malaria (≥40%), representing 18.4 million (48.2%) individuals. Of these areas, 33.3% (31/93) of IUs had ITN coverage ≥40%, of which only 9.7% (3/31) had the highest coverage. Conversely, none of the areas with high prevalence of both LF and malaria had coverage at the lowest level (<20%). IUs with high prevalence of both LF and malaria most commonly had low access (66.7% [62/93] of IUs, containing 13.3 million (72.3%) people) and low use (65.6% (61/93) of IUs, containing 12.7 million (69.0%) people).

Revised text (lines 215-221):

Approximately 53.5% (93/174) of high LF prevalence areas had high or highest prevalence of malaria, representing 18.4 million (48.2%) individuals. Of these areas, 33.3% (31/93) of IUs had both ITN access and use ≥40%, of which only 9.7% (3/31) had the highest levels of both access and use. IUs with high prevalence of both LF and malaria most commonly had low access (66.7% [62/93] of IUs, containing 13.3 million [72.3%] people) and low use (65.6% [61/93] of IUs, containing 12.7 million [69.0%] people). None of the areas with high prevalence of both LF and malaria had both access and use in the lowest categories, however.

Previous text (lines 237-239):

For the lowest ITN coverage, there were 3.9% (19/484) IUs containing 1.6 million people. In terms of high access and use, 16.7% (81/484) IUs had high ITN coverage, and 13.2% (64/484) had the highest coverage.

Revised text (lines 237-239):

Only 3.9% (19/484) of these low-LF prevalence, but high-or-highest malaria prevalence IUs, containing 1.6 million people, had the lowest ITN access and use. In comparison, 50.0% (242/484) of these IUs had high ITN access and use, and 13.2% (64/484) the highest.

I find the following sentences in the abstract very obscure and contradictory:
 "in the analysis, almost half of the locations (47.1%; 82/174 of IUs) with high LF prevalence (>5%) had at least 40% coverage with ITN access and use. Among high LF prevalence areas, both access and use were low, with 51.7% (90/174) having both access and use estimates under 40%. Additionally when classified using malaria PfPR, most (67.8%; 61/90) of these low ITN coverage, high LF prevalence locations were also considered high prevalence for malaria, Among areas with low LF prevalence (<5%), over half had ITN access >=40% (56.0%, 2812/5021) while only 48.5% (2437/5021) had use >=40%"
 I have read this several times, but still cannot grasp what you are trying to say. The problems include
 1. sometimes reporting combined access and use, and sometimes the two separately

We thank the reviewer for their comment. We have revised the results presented in the abstract as follows to increase clarity (lines 58-65):

Previous text (lines 58-65):

In this analysis, almost half of the locations (47.1%; 82/174 of IUs) with high LF prevalence (>5%) had at least 40% coverage with ITN access and use. Among high prevalence LF areas, both access and use were low, with 51.7% (90/174) of these IUs having both access and use estimates under 40%. Additionally, when classified using malaria *Pf*PR, most (67.8%; 61/90) of these low ITN coverage, high LF prevalence locations were also considered high prevalence for malaria. Among areas with low LF prevalence (<5%), over half had ITN access >=40% (56.0%; 2,812/5,021), while only 48.5% (2,437/5,021) had use >=40%.

Revised text (lines 58-65):

In this analysis, ITN coverage was low in areas where LF is common, with 51.7% (90/174) of high-LF-prevalence IUs having both ITN access and use estimates under 40%. Most (67.8%; 61/90) of these low-ITN-coverage, high-LF-prevalence locations were also categorized as high- or highest-prevalence for malaria by *Pf*PR, suggesting suboptimal ITN coverage even in some malaria-co-endemic locations. Even in IUs with high LF prevalence but low malaria prevalence, almost half (48.2%; 39/81) had high levels of access to ITNs. When accounting for population, however, gaps in ITN access in such areas were evident: more individuals lived in high-LF, low-malaria IUs with low ITN access (8.68 million) than lived in high-LF, low-malaria IUs with high ITN access (6.76 million).

We have also made revisions to the legends of Figures 1-4 (lines 181-182, 188-189, 202-204, and 208-210 respectively), and Supplementary Figures 1-10 for increased clarity as follows, with legends differing in measures referenced:

Example of previous text (Figures 1-4, Supplementary Figures 1-10):

The bivariate choropleth map and scatter plot color key in the center indicate the degree to which LF prevalence and ITN use overlap, with LF prevalence increasing as you move up the grid and ITN use increasing as you move to the righ~~t~~.

Example of revised text (Figures 1-4, Supplementary Figures 1-10):

The bivariate choropleth map and scatter plot color key in the center indicate the degree to which LF prevalence (vertical axis, white to red) and ITN use (horizontal axis, white to blue) overlap.

2. including the % cutoffs for the levels of "coverage" or prevalence (not really necessary once defined in text), next to the % in each category

Based upon this feedback, the abstract sentence in question was edited as above. In addition, we have defined the categorical levels of ITN access, use, and malaria prevalence once in the methods (lines 166-168), as indicated above in response to Reviewer 2’s first question in the Results section, and made similar edits, removing percent cutoff definitions in lines 58-60, 171-172, 195-196, 215-216, 227-231, 234-235.

3. some comparisons using IUs and some using 5km2 units.. Why?

We have edited the text to include details on IU aggregation in lines 56-58 in the abstract and lines 143-149 in the methods, as indicated above in response to Reviewer 2’s second comment, in response to the reviewer’s feedback, in order to more clearly emphasize that the primary unit of analysis in this manuscript was the implementation unit.

Previous abstract text:

We overlaid the LF prevalence estimates with ITN estimates at the 5km^2^ level for 33 LF and malaria-endemic locations in Africa.

Revised abstract text:

We aggregated the 5x5 km^2^ estimates of LF prevalence estimates and ITN estimates to the implementation unit (IU) level using fractional aggregation, for 33 LF and malaria-endemic locations in Africa, and then overlaid the IU-level aggregates.

So what do we conclude? The malaria programs have successfully covered the LF high prevalence areas, or not? Do we need special net programmes in low malaria/high LF areas?

Abstract text and the discussion have been updated on lines 58-69 and 247-250, 271-281, and 327-331, respectively, to better convey paper findings and implications for policy decisions, following the reviewer’s comment.

Previous abstract text:

In this analysis, almost half of the locations (47.1%; 82/174 of IUs) with high LF prevalence (>5%) had at least 40% coverage with ITN access and use. Among high prevalence LF areas, both access and use were low, with 51.7% (90/174) of these IUs having both access and use estimates under 40%. Additionally, when classified using malaria *Pf*PR, most (67.8%; 61/90) of these low ITN coverage, high LF prevalence locations were also considered high prevalence for malaria. Among areas with low LF prevalence (<5%), over half had ITN access >=40% (56.0%; 2,812/5,021), while only 48.5% (2,437/5,021) had use >=40%. These results illustrate the degree to which malaria control programs have achieved access to and use of ITNs in LF-endemic areas. As LF can be transmitted by multiple vector species, the impact of ITNs on LF prevalence may vary depending on the predominant vector species and their habits. Furthermore, location differences in MDA coverage, ITN implementation, and other factors will also affect ITN impact. Spatial analyses like these can be combined with other context-specific knowledge to help inform future elimination and control strategies.

Revised abstract text:

In this analysis, ITN coverage was low in areas where LF is common, with 51.7% (90/174) of high-LF-prevalence-IUs having both access and use estimates under 40%. Most (67.8%; 61/90) of these low-ITN-coverage, high-LF-prevalence locations were also categorized as high- or highest-prevalence for malaria by *Pf*PR, suggesting suboptimal ITN coverage even in some malaria-co-endemic locations. Even in IUs with high LF prevalence but low malaria prevalence, almost half (48.2%; 39/81) had high levels of access to ITNs. When accounting for population, however, gaps in ITN access in such areas were evident: more individuals lived in high-LF, low-malaria IUs with low ITN access (8.68 million) than lived in high-LF, low-malaria IUs with high ITN access (6.76 million). These results suggest that relying on current malaria vector control programs alone may not provide sufficient ITN coverage for high LF prevalence areas. Opportunities for coordinated vector control programs in places where LF and malaria prevalence are high but ITN coverage is low – or additional ITN distribution in high-LF, low-malaria locations - should be explored to help achieve elimination goals.

Additional discussion text lines 247-250:

For the high LF prevalence areas with low malaria prevalence, despite almost half of IUs having high ITN access, more individuals lived in IUs with low ITN access, suggesting that relying on current malaria vector control programs may not be sufficient for some high LF prevalence areas.

Additional discussion text lines 271-281 (additions have been underlined):

For instance, some malaria ITN programs primarily distribute ITNs via antenatal clinics, with pregnant persons and children serving as the main target populations due to high health burdens in these groups which could explain some of the low ITN coverage in high malaria prevalence areas observed in our findings (34). For LF, these strategies may be suboptimal, given that preventing infection across the lifespan is of particular importance to prevent the disabling sequelae of chronic and repeated parasite exposure (35,36). In these locations, collaboration to extend the scope of the ITN program could be considered to extend benefits to LF control programs.

The partial overlap seen in this analysis of areas with high LF prevalence and high malaria prevalence could indicate that LF programs may need to consider alternative ITN distribution methods or special net programs for high-LF but low-malaria locations with low ITN coverage, such as some of those outlined in the WHO’s document on scaling up ITNs (34).

Additional discussion text lines 327-331:

Where the predominant vector species distributions and the context of MDA and other control efforts suggest a role for ITNs in LF control and elimination, these results help to identify locations where additional ITN coverage may be of the most benefit for both diseases. In high-LF, low-malaria locations with low ITN coverage, LF-driven programs to enhance ITN coverage may be needed.

It might help to provide some tables on the distribution of the geographical units into different categories of LF prevalence and ITN access/use, stratified by malaria PfPR.

We thank the reviewer for their suggestion. Please see the new table in the supplementary appendix (S1 Table), also referenced on lines 240-241. When adding the new table we also made corrections to results presented on lines 229-231, and 234-239.

Previous text lines 229-231:

There were 509 million (56.4%) individuals living in IUs with low LF prevalence (<5%) but with ITN coverage ≥40% (56.0% of IUs; 2,812/5,021), compared to 124 million people (13.7%) living in IUs with the highest ITN coverage (19.1%; 961/5,021 IUs)

Corrected text lines 229-231 (changes underlined):

There were 509 million (56.4%) individuals living in IUs with low LF prevalence (<5%) but with ITN access ≥40% (56.0% of IUs; 2,812/5,021), compared to 124 million people (13.7%) living in IUs with the highest ITN access (19.1%; 961/5,021 IUs)

Previous text lines 234-239:

Within these, there were 18.4 million (21.8%) individuals living in IUs with low ITN access (21.7%; 105/484 IUs) and 14.0 million individuals in IUs with low ITN use (16.9%; 82/484 IUs). For the lowest ITN coverage, there were 3.9% (19/484) IUs containing 1.6 million people. In terms of high and highest access and use, 16.7% (81/484) IUs had high ITN coverage, and 13.2% (64/484) had the highest coverage.

Corrected text lines 234-239 (changes underlined):

Within these, 18.4 million (21.8%) individuals lived in IUs with low ITN use (21.7%; 105/484 IUs) and 14.0 million individuals in IUs with low ITN access (16.9%; 82/484 IUs). Only 3.9% (19/484) of these low-LF prevalence, but high-or-highest malaria prevalence IUs, containing 1.6 million people, had the lowest ITN access and use. In comparison, 50.0% (242/484) of these IUs had high ITN access and use, and 13.2% (64/484) the highest.

I don't see why the LF prevalence count maps are useful (figs 1 and 2) in addition to the LF prevalence maps, if the counts are not adjusted for population. What do they add?

We thank the reviewer for their comment and for the opportunity to expand upon this in the text. We have added information regarding the population estimates used into the methods on lines 145-151, as indicated above in response to Reviewer 2’s second comment. The count maps do account for population. The maps included give two important views into the relationship between LF and ITN access: the prevalence vs ITN coverage maps show where the IUs are with the highest proportion of people having LF, and what ITN coverage is like there. The count-based maps, however, help to highlight the places where the most people are living with LF. Policymakers may be interested in both lenses when making prioritization decisions: the count-based framework would conceptually help to identify the places where the largest number of people could benefit from interventions like enhanced ITN coverage, while the prevalence-based framework helps to identify locations where the relative LF burden is likely to be highest, which is particularly important for understanding and mitigating within-country inequalities.

In the Discussion, the first paragraph is very important, and should be captured better in abstract somehow. But I am puzzled by the last sentence (and lines 248 to 250 and lines 259 to 261 which make the same point). If half of the areas had high malaria prevalence, then wouldn't they be targeted for ITN distribution anyway?. If they have low ITN access and use, doesn't that represent a failure of the malaria programme, not need for integration or extra programmes? The areas of most concern would seem to me to be those with low malaria but high LF, where special effort to provide nets for LF might be needed.

We thank the reviewer for their comment and agree that in some places, ITN coverage in malaria-endemic locations is suboptimal, which we allude to in the discussion (lines 246-247). As mentioned in the text, in some countries, malaria ITN programs are focused on only a subset of the population (lines 271-277). In these cases, access to ITNs in the general population may be low even if the access in those specific populations may be high. This wouldn’t indicate a failure of the malaria programs, but rather a difference in scope and intent of the programs – and a potential opportunity for the two programs to collaborate going forward, particularly if broader coverage for LF control is desired. We have revised the discussion to better highlight these different scenarios, as indicated above in response to Reviewer #2, the question regarding conclusions made (lines 247-250, 271-281, and 327-331), to address this comment. We have also made similar changes in other locations in the manuscript: lines 58-61 in the abstract (also addressed above), 222-226 in the results, and 247-250 in the discussion.

Additional results text (lines 222-226):

Of the IUs with high LF prevalence but low malaria prevalence, almost half had high ITN access (48.2%; 39/81) and over half had high ITN use (54.3%; 44/81) representing 6.76 million and 8.08 million individuals respectively. Even though only around one third of IUs (34.6%; 28/81) had low ITN access, there were more people living in these IUs (8.68 million) than in those with high ITN access. These low access IUs were primarily located in Nigeria.

Additional discussion text (lines 247-250):

For the high LF prevalence areas with low malaria prevalence, despite almost half of IUs having high ITN access, more individuals lived in IUs with low ITN access, suggesting that relying on current malaria vector control programs may not be sufficient for some high LF prevalence areas.

**Conclusions**


-Are the conclusions supported by the data presented?

-Are the limitations of analysis clearly described?

-Do the authors discuss how these data can be helpful to advance our understanding of the topic under study?

-Is public health relevance addressed?

Reviewer #1: Yes

We thank the reviewer for their kind comment.

Reviewer #2: The last three sentences of the abstract are very general, and could have been written before the work was done. They do not summarize the findings of the study. What can we conclude based on the data presented here? What is the degree to which malaria control programmes have achieved access and use to ITNs in malaria endemic areas? What more should programmes be doing? In which areas should net distribution be enhanced (line 79?).

We thank the reviewer for their critique. We have revised the abstract based on this feedback (lines 65-69), as indicated above in response to Reviewer #2’s question regarding conclusions made.

If net access/use is low in high malaria and LF areas, isn't that a failure of net distribution for malaria, not a need for 'integration' whatever that means.

We have addressed this under the similar question made above.

**Editorial and Data Presentation Modifications?**

Use this section for editorial suggestions as well as relatively minor modifications of existing data that would enhance clarity. If the only modifications needed are minor and/or editorial, you may wish to recommend “Minor Revision” or “Accept”.

Reviewer #1: I am not sure if the addition of published manuscripts as part of the supplementary materials is important. The reference to these manuscripts should suffice.

We thank the reviewer for their comment, and the manuscripts in the supplementary materials have been removed.

Reviewer #2: Lines 94 to 96 are not quite correct. Post-validation surveillance (PVS) is not the period of transmission assessment after MDA - that is post MDA surveillance, which is first. PVS comes after validation (several years after MDA stops and multiple TAS surveys have been done)

We thank the reviewer for pointing this out. We have amended the text at lines 93-97.

Previous text:

Under the World Health Organization (WHO)–established Global Programme to Eliminate Lymphatic Filariasis (GPELF), many countries have made significant progress: 17 countries have entered post-validation surveillance (period of transmission assessment after mass drug administration [MDA]), 11 have reached post-MDA surveillance, and all but two remaining countries have delivered MDA in some capacity (1).

Revised text:

Under the World Health Organization (WHO)–established Global Programme to Eliminate Lymphatic Filariasis (GPELF), many countries have made significant progress: 17 countries have entered post-validation surveillance (ongoing transmission monitoring following GPELF certification recognizing elimination of LF as a public health problem), 11 have reached post–mass drug administration (MDA) surveillance, and all but two remaining countries have delivered MDA in some capacity (1).

A couple of places with missing refs line 195 and 225

We thank the review for identifying this; the missing references were added.

Results text 163-171, 190-195 and 201 onwards, consider putting into Tables to make clearer. It's quite hard to read at the moment.

We thank the reviewer for this suggestion and have added a table of results to the supplementary files (S1 Table, referenced in lines 240-241).

The Discussion seems overlong, while the Results section could be expanded to be clearer. The limitations of the data analysis are well described, but a bit belaboured since the work is quite comprehensive and impressive.

We thank the reviewer for their kind comments and appreciate their feedback regarding discussion length. We have edited the discussion in response to the reviewers’ comments, which resulted in both revisions to the existing text and the addition of several new discussion points. We have also restructured part of the limitations section to focus on key limitations, while moving comparisons to other studies to an earlier part of the discussion (lines 251-254). We would be happy to take any additional reviewer or editorial guidance on the structure, length, and content of the discussion.

Previous limitations section:

This analysis carries some limitations. We chose to compare the most recent available geospatial estimates for LF prevalence, ITN coverage, and malaria prevalence, in order to produce the most up-to-date comparisons possible at the time of analysis. As LF results were only available through the year 2018, however, these results do not fully reflect any recent changes in the current spread and level of LF prevalence. Furthermore, both LF and malaria estimates may be subject to accuracy limitations where data is sparse. As mentioned, this analysis used malaria ITN estimates among the total population and does not account for any ITN distribution that may have happened outside of malaria vector control initiatives. An analysis of ITN coverage and LF prevalence over time was outside the scope of this paper, however, as has been seen in some country-specific analyses (20)., such an analysis could provide important context on historical trends and progress and is planned for the future once prevalence estimates are able to be updated with more recent data. There have been other studies looking at LF prevalence and ITN coverage as well as LF prevalence and malaria prevalence; however, these have primarily been country-specific analyses and only cover a subset of endemic African countries (35,52,66–68). Although a direct comparison with these analyses is not likely to be informative due to estimates being from different years, it is important to note that some country-specific trends seen in this analysis, such as ITN coverage being generally low in areas where LF prevalence was high and partial overlap of high LF and malaria prevalence areas, echo some the findings of these other studies (66,67). To the authors’ knowledge this is one of the first papers looking at the overlap of LF and ITNs across endemic Africa.

Revised limitations section:

This analysis carries some limitations. We chose to compare the most recent available geospatial estimates for LF prevalence, ITN coverage, and malaria prevalence, in order to produce the most up-to-date comparisons possible at the time of analysis. As LF results were only available through the year 2018, however, these results do not fully reflect any recent changes in the current spread and level of LF prevalence. Furthermore, both LF and malaria estimates may be subject to accuracy limitations where data is sparse. As mentioned, this analysis used malaria ITN estimates among the total population and does not account for any ITN distribution that may have happened outside of malaria vector control initiatives. An analysis of ITN coverage and LF prevalence over time was outside the scope of this paper, though has been examined in previous country-specific analyses (20).

Revised study comparison text (lines 251-254):

Previous studies evaluating the intersection of LF prevalence, ITN coverage, and malaria prevalence have primarily been limited to a subset of endemic African countries (20–24). Several of the findings from this analysis, such as ITN coverage being generally low in areas where LF prevalence was high and partial overlap of high LF and malaria prevalence areas, echo the results of these other studies (21,23).

**Summary and General Comments**

Use this section to provide overall comments, discuss strengths/weaknesses of the study, novelty, significance, general execution and scholarship. You may also include additional comments for the author, including concerns about dual publication, research ethics, or publication ethics. If requesting major revision, please articulate the new experiments that are needed.

Reviewer #1: The paper describes the overlap of LF, malaria, and ITN (access and use) and is important in understanding the role of vector control for LF. Although I am unable to assess the statistical approaches, the results are informative. Overall, the paper is well written. There are just some few comments that I believe the authors should address.

Line 55: Italicise Plasmodium falciparum
 Line 121: delete the word "endemic" before Africa.
 Lines 157 - 158: "For ITN coverage (access and use) and malaria prevalence we used the following definitions: lowest <20%; low 20-39.9%; high 40%-59.9%; highest ≥60%" Kindly clarify. Are these thresholds for ITN coverage or malaria prevalence?
 Line 167: Check the reference error
 Line 195: Chech the reference error
 Line 225: Check the reference error

We thank the reviewer for the complimentary comments. Text edits were made per reviewer’s suggestions.

- An important question to be addressed in this work is the ITN coverage level that results in significant impact on LF prevalence and control. ITN access and use >40% does not really explain the level at which ITNs start having an impact. In Figures 1 and 2, for instance, areas with high LF and high ITN access are shown. However, if high ITN access and use was effective, one would expect low LF prevalence in these high ITN access and use areas. How do the authors explain these?

We thank the reviewer for this feedback. While ITNs are an important tool that can contribute to accelerating elimination and eradication programs (lines 287-289), their impact is influenced by multiple factors, such as the predominant vector species and their habits (lines 291-294). Therefore, it is important to consider them as only one part of the full package of LF control measures (lines 296-307). We agree that subsequent studies to estimate the relative contribution of all of these control measures in determining LF prevalence - and how those contributions vary from place to place - would be extremely valuable but are outside the scope of this analysis.

Lines 273 - 283: In parts of Africa, especially along the East Africa coast, Culex species play a role in the transmission of LF. The authors should explain the overlap of high LF and high malaria in these areas and possibly the low impact of ITNs.

- It is also important to note that malaria vector interventions result in vector behavior modifications, with vectors biting more outdoors than indoors. This could possibly explain the lack of correlation between high malaria and high ITN use and high LF prevalence and should be discussed.

We discuss insecticide resistance for areas with *Culex* or *Anopheles* as primary vectors in lines 308-309. Differential biting habits are mentioned in lines 291-294 as well. We agree with the reviewer that this additional interaction is important to note, and have added in text regarding vector behavior modification on lines 294-295 to better highlight this important point:

Additional text:

Vector control programs also have the potential to drive vector behavior modification which may lead to decreased ITN impact (49-51).

Reviewer #2: Overall, this is a good piece of work combining these datasets but it needs more clarity in the results and more interpretation of what the findings mean for programmes.

We thank the reviewer for their kind comments and appreciate the opportunity to strengthen this manuscript through revision based upon their feedback.
