# Supplementary material for: Examining the overlap in lymphatic filariasis prevalence and malaria insecticide-treated net access-use in endemic Africa
Source: PLoS Negl Trop Dis. 2025 Jun 26;19(6):e0013165. doi: 10.1371/journal.pntd.0013165 (PMC12201664; doi:10.1371/journal.pntd.0013165)
Supplement: Attachment — Submitted filename: Second round Response to Reviewers.docx [file pntd.0013165.s015.docx]

PLOS NTDs paper revisions, 2^nd^ round 2025-03-25

Author comments are in blue.

Line numbers referenced refer to the lines in the tracked changes version of the manuscript once all revisions have been accepted.

**Methods**

-Are the objectives of the study clearly articulated with a clear testable hypothesis stated?

-Is the study design appropriate to address the stated objectives?

-Is the population clearly described and appropriate for the hypothesis being tested?

-Is the sample size sufficient to ensure adequate power to address the hypothesis being tested?

-Were correct statistical analysis used to support conclusions?

-Are there concerns about ethical or regulatory requirements being met?

Reviewer #1: Yes

Reviewer #2: Thanks for the diligence in responding to the comments. I am happy with the responses relevant to this section.

We thank both reviewers for their kind comments.

**Results**

-Does the analysis presented match the analysis plan?

-Are the results clearly and completely presented?

-Are the figures (Tables, Images) of sufficient quality for clarity?

Reviewer #1: Yes

We thank the reviewer for their comment.

Reviewer #2: Thanks for providing the new Table classifying IUs as suggested. It is very useful to be able to conceptualize the scale of the problem and should help program managers. I strongly suggest that it be put into the main text as most people don't look at Supplementary files and this table is very important and useful.

In response to the reviewer’s suggestion we have moved Table 1 to the main text. Please see line 171 for the updated in-text reference, and lines 179-181 for the Table.

Removed text (lines 242-243):

Table in S1 Table shows the distribution of geographical units by LF prevalence, ITN access, and ITN use, stratified by malaria *Pf*PR.

Added in-text reference (lines 170-172; addition underlined):

In this analysis, although only 3.4% (174/5,195) of IUs were categorized as having high LF prevalence (Table 1), a total of 38.2 million individuals lived in these locations, primarily located in Nigeria, Côte d’Ivoire, and Liberia.

**Conclusions**

-Are the conclusions supported by the data presented?

-Are the limitations of analysis clearly described?

-Do the authors discuss how these data can be helpful to advance our understanding of the topic under study?

-Is public health relevance addressed?

Reviewer #1: Yes

Reviewer #2: Revised text is much better and clearer.

We thank both reviewers for their complimentary comments.

**Editorial and Data Presentation Modifications?**


Use this section for editorial suggestions as well as relatively minor modifications of existing data that would enhance clarity. If the only modifications needed are minor and/or editorial, you may wish to recommend “Minor Revision” or “Accept”.

Reviewer #1: (No Response)

Reviewer #2: Ref 19 is a bit garbled (Organization, WH Filariasis GP to EL?). Can be solved in EndNote etc by where you put the comma. WHO should probably be listed as author in several other refs e.g. 1, 2,7, 10, 11, 34, 57.

Something missing ref 53? Biology and control of....

We thank the reviewer for pointing these out, and we apologize for the formatting and reference issues identified here. It appears that these issues resulted from a corrupted link between our citation manager and the manuscript document. We have now reviewed all of the references, including those mentioned, and have made updates as needed.

**Summary and General Comments**


Use this section to provide overall comments, discuss strengths/weaknesses of the study, novelty, significance, general execution and scholarship. You may also include additional comments for the author, including concerns about dual publication, research ethics, or publication ethics. If requesting major revision, please articulate the new experiments that are needed.

Reviewer #1: My comments are appropriately addressed.

We thank the reviewer for their kind comment.

Reviewer #2: Overall the authors have been very responsive to the comments and the paper seems much improved as a result.
 I have two remaining comments, in addition to suggesting that the new Supp Table be put in main text:

Lines 271-281 about net distribution policies, alluded to a couple of times in the responses: It is true that in the early days of LLIN, nets were prioritized to children under 5 and pregnant women through antenatal clinics. This happened quite a long time ago (>10 yrs) and I am not aware of anywhere that uses this as the sole method now as it is known not to achieve high enough access (it is used a supplementary continuous distribution method in some countries). So I would change this text to refer to past distribution policies, which may indeed have resulted in low access, but policies have already moved on in WHO and other guidelines towards universal (all ages in population) coverage (could be cited [https://www.who.int/publications/i/item/guidelines-for-malaria](https://urldefense.com/v3/__https:/www.who.int/publications/i/item/guidelines-for-malaria__;!!K-Hz7m0Vt54!j96bzSzV09iej_qR53LdP0-F34_YyvzsdMS05OrYPvtKPpJfWriUk0gEXLESi-y8ZDVFYg6OjAe7o7k$)) and followed by Global Fund etc . Gaps now are probably more about lack of sub-national prioritization of universal coverage in highest risk areas rather than blanket national policies. You could say "PAST sub-optimal ITN coverage" and "IN THE PAST, some malaria ITN programs primarily distributed ITNs via antenatal clinics". "Extend the scope of ITN programmes" is a bit vague and might be better as "increase the access and use of nets in the highest priority areas" or similar.

We thank the reviewer for their comments and appreciate their feedback regarding distribution policies. We have revised the text in response to the reviewer’s comments on lines 273-282.

Previous text:

For instance, some malaria ITN programs primarily distribute ITNs via antenatal clinics, with pregnant persons and children serving as the main target populations due to high health burdens in these groups which could explain some of the low ITN coverage in high malaria prevalence areas observed in our findings (34). For LF, these strategies may be suboptimal, given that preventing infection across the lifespan is of particular importance to prevent the disabling sequelae of chronic and repeated parasite exposure (35,36). In these locations, collaboration to extend the scope of the ITN program could be considered to extend benefits to LF control programs.

Revised text:

In the past, some malaria ITN programs primarily distributed ITNs via antenatal clinics and Expanded Programme for Immunization (EPI) visits, with pregnant persons and children serving as the main target populations due to high health burdens in these groups (34). For LF, these strategies may have been suboptimal, given that preventing infection across the lifespan is of particular importance to prevent the disabling sequelae of chronic and repeated parasite exposure (35,36). Although antenatal and EPI visits still play an important role in some countries for continuous ITN distribution (37), since 2007 the WHO has recommended a shift in distribution strategy towards universal coverage (10,37), and more recently towards the subnational tailoring of interventions (38). In alignment with these recommendations, collaboration to increase the access and use of nets in the highest priority areas could be considered to extend benefits to LF control programs.

In the paragraph lines 287 to 297 you do not currently cite the paper by Richards et al 2013 Community-Wide Distribution of Long-Lasting Insecticidal Nets Can Halt Transmission of Lymphatic Filariasis in Southeastern Nigeria
 [https://doi.org/10.4269/ajtmh.12-0775](https://urldefense.com/v3/__https:/doi.org/10.4269/ajtmh.12-0775__;!!K-Hz7m0Vt54!j96bzSzV09iej_qR53LdP0-F34_YyvzsdMS05OrYPvtKPpJfWriUk0gEXLESi-y8ZDVFYg6OPKavo9Y$). This is a very relevant paper you could consider citing that shows the impact of LLIN on LF transmission in an area in Africa, with multiple LF vectors, that had not had MDA for LF, comparing full access of nets with targeted (under five/pregnant women) distribution. An important finding apart from the impact on LF transmission was how many nets had to be given out to get full access (1 net per 2 people) in many large households. Disclosure: I co-managed this project and analysis, and am an author on the paper .

We appreciate the reviewer bringing this important paper to our attention. We have added additional text highlighting this paper’s findings on lines 292-306 and added it as reference on line 294.

Previous text:

While vector control is not currently required for validation of EPHP, there is evidence that it helps to greatly reduce LF prevalence in some settings and could accelerate elimination and eradication programs (8,22,38-43). A study in Papua New Guinea found that the introduction of ITNs directly led to a decrease in the annual infective biting rate (40), and in The Gambia, LF elimination was reached in the absence of MDA while scaling up ITNs (44). However, as LF can be transmitted by multiple vector species, the impact of ITNs on LF prevalence is likely to vary depending on the predominant vector species and their habits, such as whether the species tends to feed outdoors (exophagic) or indoors (endophagic) and when peak biting times occur (8,43,45,46). Vector control programs also have the potential to drive vector behavior modification which may lead to decreased ITN impact (47-49). Furthermore, location differences in MDA coverage, ITN implementation, IRS use, and other factors will also affect ITN impact.

Revised text:

While vector control is not currently required for validation of EPHP, there is evidence that it helps to greatly reduce LF prevalence in some settings and could accelerate elimination and eradication programs (8,22,40-46). A study in Papua New Guinea found that the introduction of ITNs directly led to a decrease in the annual infective biting rate (42), and in The Gambia, LF elimination was reached in the absence of MDA while scaling up ITNs (47). However, as LF can be transmitted by multiple vector species, the impact of ITNs on LF prevalence is likely to vary depending on the predominant vector species and their habits, such as whether the species tends to feed outdoors (exophagic) or indoors (endophagic) and when peak biting times occur (8,45,48,49). Encouragingly, a study conducted in an area of Southeastern Nigeria endemic for both malaria and LF that had not undergone MDA due to co-endemicity with *Loa loa* showed that, even in the presence of multiple LF vectors and high transmission, LF transmission could be halted with the use of ITNs alone – though coverage of 1 net per 2 people in each household was required to do so (46). Vector control programs also have the potential to drive vector behavior modification which may lead to decreased ITN impact over time (50-52). Furthermore, location differences in MDA coverage, ITN implementation, IRS use, and other factors will also affect ITN impact.

The other responses are comprehensive and satisfactory, thanks.

We thank the reviewer for their kind comment.
